# Case Report: Non-convulsive seizure following traumatic brain injury — a significant occurrence that needs to be considered due to potential long-term sequelae

**DOI:** 10.12688/f1000research.135482.1

**Published:** 2023-09-14

**Authors:** Azra Zafar

**Affiliations:** 1 Department of Neurology, College of Medicine, Imam Abdulrahman Bin Faisal University, Dammam, Eastern Province, Saudi Arabia

**Keywords:** Nonconvulsive seizure; diffuse axonal injury; temporal lobe; excitotoxicity; nonconvulsive status epilepticus

## Abstract

Introduction/background

Non convulsive seizures (NCS) following traumatic brain injury (TBI) may remain undiagnosed due to lack of overt clinical manifestation and can have long-term sequelae due to delay in timely treatment. Occurrence of early NCS is known to have subsequent neurologic sequelae due to excitotoxic neuronal injury.

Case report

This is a case report of a young girl who sustained a TBI due to a motor vehicle accident (MVA) and was admitted with a fluctuating level of consciousness. Her clinical presentation was attributed to TBI; however as her conscious level did not recover, an electroencephalogram (EEG) was requested, which detected non convulsive status epilepticus (NCSE). Anti-seizure medication (ASM) was started. Her follow-up EEG and magnetic resonance imaging (MRI) were suggestive of the potential adverse effects of prolonged NCSE.

Conclusion

NCS may remain undiagnosed in TBI due to a paucity of overt clinical manifestations. Every patient with TBI and altered consciousness at presentation should be evaluated by EEG immediately, if possible, in the emergency department to avoid long-term sequelae of NCS in such cases.

## Case report

A 13-year-old, medically free student was brought to the King Fahd Hospital of the University (KFHU) after sustaining a motor vehicle accident (MVA) as a car passenger. Due to the side impact, the patient did not sustain any open injuries, and did not eject from the car. No evidence of vomiting, convulsions, nose or ear bleeding was present upon arrival either. However, her consciousness level was fluctuating with intermittent episodes of agitation. On examination, she was drowsy. Her vitals were within normal limits and her Glasgow Coma Scale score was 10/15. Neurological examination did not detect any focal abnormality except a bilateral positive Babinski sign.

**Figure 1.**
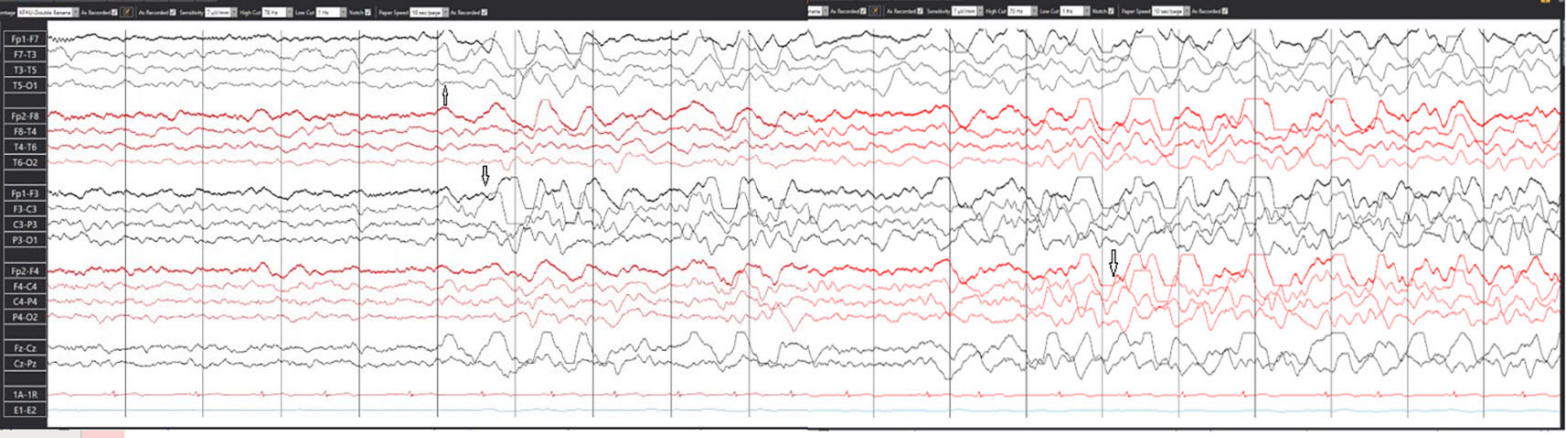
EEG showing electrographic seizure.

Routine blood works and pan body computed tomography scan were normal. Once the patient was admitted to the intensive care unit (ICU), an EEG was requested to rule out non convulsive seizure (NCS) for unexplained altered sensorium. Her first EEG was performed 65 hrs after admission. It detected encephalopathy and electrographic seizures (ESz) arising from the left anterior temporal region ( Figure 1), indicating non-convulsive status epilepticus (NCSE). Levetiracetam (LVT) was initiated, and an MRI scan was subsequently performed which revealed multiple hyperintense foci in bilateral frontal and right temporal and occipital lobe, as well as hemorrhage foci on susceptibility weighted image (SWI). These findings were indicative of hemorrhagic axonal diffuse injury type (DAI) type 2. Despite starting LVT, the patient’s consciousness level did not improve; therefore, EEG was repeated after 24 hrs, indicating that NCSE had not been resolved. At this stage, phenytoin was added, after which the patient’s consciousness level improved. The follow-up EEG showed focal epileptic discharges in the form of sharp waves and spikes in the left centrotemporal head region. Patient was discharged on two anti-seizure medications (ASM).

## Follow-up diagnostic investigations

**Figure 2.**
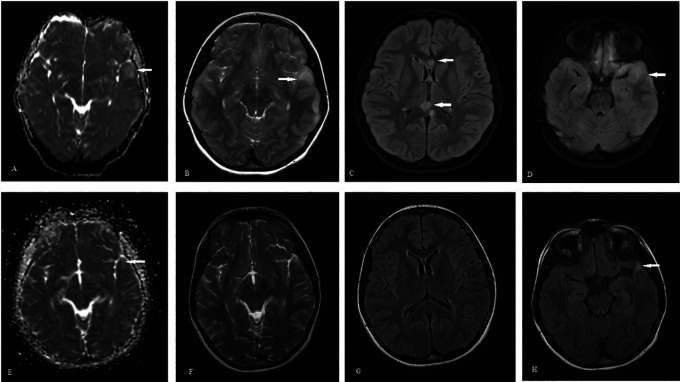
A-D: Initial MRI brain images showing changes of diffuse axonal brain injury; A - ADC sequence showing edema, B - T2 weighted images showing hyperintense left temporal lobe, C - FLAIR sequence showing hyperintense signals in corpus callosum, D - FLAIR sequence showing hyperintense left temporal lobe suggestive of edema. E-H: Follow up MRI brain images; E - ADC sequence showing residual minimal bright signal in left temporal area, F - T2 weighted sequence showing significant resolution of left temporal edema, G - FLAIR image showing resolution of changes in corpus callosum, H - FLAIR sequence showing residual hyperintense lesion in left temporal lobe.

**Figure 3.**
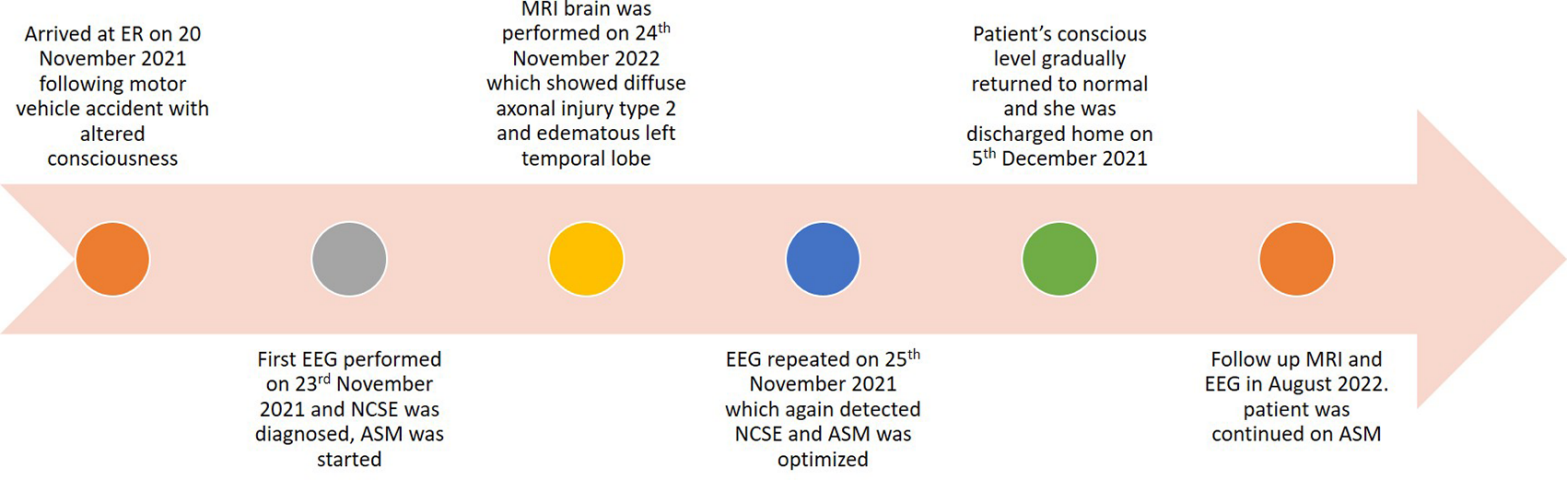
Time line of case.

During her follow-up visits, she was symptoms free. Brain MRI and EEG were repeated nine months after the event to determine if ASM should be discontinued. EEG detected focal epileptic discharges in the left temporal head region, and brain MRI showed significant resolution of changes, except an abnormal focal hyperintense lesion in the left temporal area (see Figure 2). Consequently, LVT was continued (the entire timeline is summarized in Figure 3).

## Outcome

One year follow-up, the patient was seizure-free with single ASM and had no symptoms suggestive of cognitive disturbance. She was active in her normal routine life including academics.

## Discussion

TBI is a significant cause of preventable deaths in Saudi Arabia, and 95% are attributed to MVA. ^1^ Seizures (including NCS/NCSE) can occur in 20−30% of patients with severe TBI due to cerebral metabolic distress and hippocampal atrophy, which contribute to higher mortality rates. ^2^ However, in clinical practice, NCS following TBI may remain undiagnosed due to the lack of overt clinical manifestation and adversely affect outcomes due to the delay in treatment. ^3^ Occurrence of early NCS can have subsequent neurologic sequelae due to excitotoxic neuronal injury aggravating the injury caused by TBI. ^4^ Therefore, it is mandatory to diagnose NCSE in a timely manner to prevent significant neurological sequelae by performing EEG in TBI victims with altered sensorium. The benefits of this protocol are supported by the findings yielded by a study involving continuous EEG monitoring in 16 patients with severe TBI, allowing NCS to be detected in three cases. ^5^ Similarly, seizures were detected using EEG in 20% of examined patients, more than 50% of whom experienced NCS. ^6^

Our patient had TBI of moderate severity ^7^ and prolonged NCSE which lasted for five days. Brain MRI showed changes related to prolonged NCS, along with DAI. The follow-up EEG detected focal ED in the left temporal region and MRI showed a small localized hyperintense lesion in the left temporal lobe which could be either a sequela of TBI or NCSE. However, the left temporal cortical edema, which could be the result of prolonged NCS, was completely resolved. The relationship between prolonged NCS and structural changes to the brain, particularly the temporal lobe, has been reported previously. ^2^^,^^8^ Vespa et al. examined cEEG findings of 140 patients with moderate to severe TBI, and detected acute post-traumatic NCS in 23% of the cohort. Moreover, in a selected group of patients, this finding was significantly associated with long-term hippocampal atrophy. ^2^ In addition, according to one case report, a patient with schizophrenia and NCSE having increased hippocampal volume in an acute setting was later found to have hippocampal atrophy. ^8^ Although NCSE is not uncommon following TBI, its association with anatomical changes leading to hippocampal atrophy in the long term is debatable, given that significant neuronal damage due to diffuse injury itself can be a cause. Thus, further research is required to better understand these phenomena. ^9^ Jorge et al., studied 37 patients with closed head injury and concluded that hippocampal volumes were significantly lower in patients with moderate to severe head injury than in patients with mild TBI. ^10^

In our patient, follow-up brain MRI showed a significant resolution of findings detected in the initial scan; however, a small abnormal hyperintense signal was persistent in the left anterior temporal region. As volumetric MRI is not available in our healthcare facility, we were unable to assess the volume loss. Still, we posit that—in addition to TBI—prolong NCSE in our patient could be a contributing factor for this finding on follow-up MRI. Although our patient did not have any clinical seizures, follow-up EEG and MRI performed nine months after the initial incident were suggestive of temporal lobe pathology with a heightened risk of temporal lobe-onset seizure. Accordingly, her ASM could not be discontinued. In reporting on this case, our aim is to highlight the importance of close EEG monitoring in patients with TBI in whom consciousness is altered. In this particular case, brain MRI was instrumental for detecting changes not only in DAI but possibly NCS as well.

This case highlights our limitation of immediate EEG recording in patients with TBI upon arrival. The strength is the identification of uncommon electrographic patterns suggesting NCS during routine EEG recording guiding proper management.

## Conclusion

This case illustrates the possible association of prolonged NCSE following TBI with temporal lobe structural changes. It further emphasizes the need for immediate EEG monitoring in patients with TBI that present with altered sensorium. All institutes dealing with trauma cases should thus have the resources needed for emergency EEG monitoring to avoid neurological sequelae of NCS, which may otherwise remain undiagnosed.

## Consent

We confirm that we have obtained permission from the patients’ father to use images and data included in this article.

## Data Availability

All data underlying the results are available as part of the article and no additional source data are required.
