# Case Report: Non-convulsive seizure following traumatic brain injury — a significant occurrence that needs to be considered due to potential long-term sequelae

**DOI:** 10.12688/f1000research.135482.2

**Published:** 2024-01-24

**Authors:** Azra Zafar

**Affiliations:** 1 Department of Neurology, College of Medicine, Imam Abdulrahman Bin Faisal University, Dammam, Eastern Province, Saudi Arabia

**Keywords:** Nonconvulsive seizure; diffuse axonal injury; temporal lobe; excitotoxicity; nonconvulsive status epilepticus

## Abstract

**Introduction/background:**

Non convulsive seizures (NCS) following traumatic brain injury (TBI) may remain undiagnosed due to lack of overt clinical manifestation and can have long-term sequelae due to delay in timely treatment. Occurrence of early NCS is known to have subsequent neurologic sequelae due to excitotoxic neuronal injury.

**Case report:**

This is a case report of a young girl who sustained a TBI due to a motor vehicle accident (MVA) and was admitted with a fluctuating level of consciousness. Her clinical presentation was attributed to TBI; however as her conscious level did not recover, an electroencephalogram (EEG) was requested, which detected non convulsive status epilepticus (NCSE). Anti-seizure medication (ASM) was started. Her follow-up EEG and magnetic resonance imaging (MRI) were suggestive of the potential adverse effects of prolonged NCSE.

**Conclusion:**

NCS may remain undiagnosed in TBI due to a paucity of overt clinical manifestations. Every patient with TBI and altered consciousness at presentation should be evaluated by continuous EEG monitoring immediately, if possible, in the emergency department to avoid long-term sequelae of NCS in such cases.

## Case report

A 13-year-old student, without any prior medical illnesses was brought to the King Fahd Hospital of the University (KFHU) after sustaining a motor vehicle accident (MVA) as a car passenger. Due to the side impact, the patient did not sustain any open injuries, and did not eject from the car. No evidence of vomiting, convulsions, nose or ear bleeding was present upon arrival either. However, her consciousness level was fluctuating with intermittent episodes of agitation. On examination, she was drowsy. Her vitals were within normal limits and her Glasgow Coma Scale score was 10/15. The patient was agitated with a fluctuating level of consciousness at the time of presentation and did not follow commands. Cranial nerves were intact. Fundus examination was attempted but failed as patient did not cooperate due to her clinical state. Tone, power and deep tendon reflexes were normal. There were no signs of meningism. Neurological examination did not detect any focal abnormality except a bilateral positive Babinski sign.

**Figure 1.**
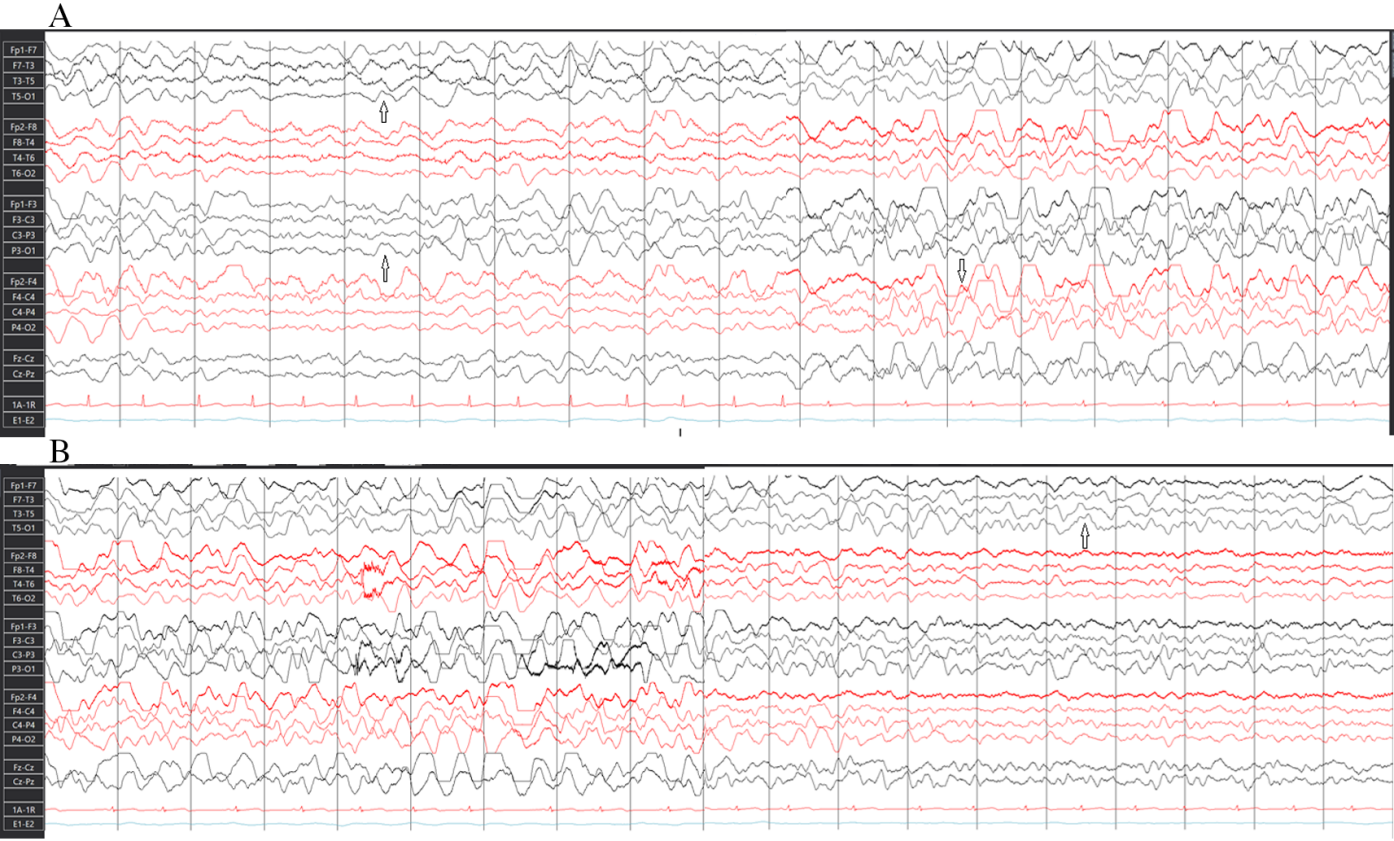
EEG showing electrographic seizure. A) Beginning of electrographic seizure; rhythmic slow waves showing propagation to right cerebral hemisphere. B) Continuation and termination of electrographic seizure.

Routine blood works and pan body computed tomography scan were normal. Once the patient was admitted to the intensive care unit (ICU), an EEG was requested to rule out non convulsive seizure (NCS) for unexplained altered sensorium. Her first EEG was performed 65 hrs after admission. It detected encephalopathy and electrographic seizures (Esz) arising from the left cerebral hemisphere ( Figure 1), indicating non-convulsive status epilepticus (NCSE). Frequent 2-3 Hz left hemispheric ictal delta activity with spatio-temporal evolution was recorded. The activity starting from the left hemisphere propagated after a few seconds to involve the right hemisphere. In, a 30-minute EEG recording, 7 events lasting for 7-20 seconds were recorded .Sharp wave activity was also recorded over the left fronto-central and temporal head region. Some spells of these epileptiform patterns were associated with subtle clinical signs which were left-hand automatism and screaming indicating electro-clinical seizures. Salzburg Consensus Criteria ^1^ were applied to diagnose NCSE.

Levetiracetam (LVT) was initiated, and an MRI scan was subsequently performed which revealed multiple hyperintense foci in bilateral frontal and left temporal and occipital lobe, as well as hemorrhage foci on susceptibility weighted image (SWI). These findings were indicative of hemorrhagic axonal diffuse injury type (DAI) type 2. Despite starting LVT, the patient’s consciousness level did not improve; therefore, EEG was repeated after 24 hrs, indicating that NCSE had not been resolved. At this stage, phenytoin was added. After starting the second ASM, the patient’s consciousness improved and returned to baseline. Patient became oriented to time, place, and person and had intact language functions. Patient was able to walk independently and had normal motor, cerebellar, and sensory system examination before discharge. The follow-up EEG showed focal epileptic discharges in the form of sharp waves and spikes in the left temporal head region. Patient was discharged on two anti-seizure medications (ASM).

## Follow-up diagnostic investigations

**Figure 2.**
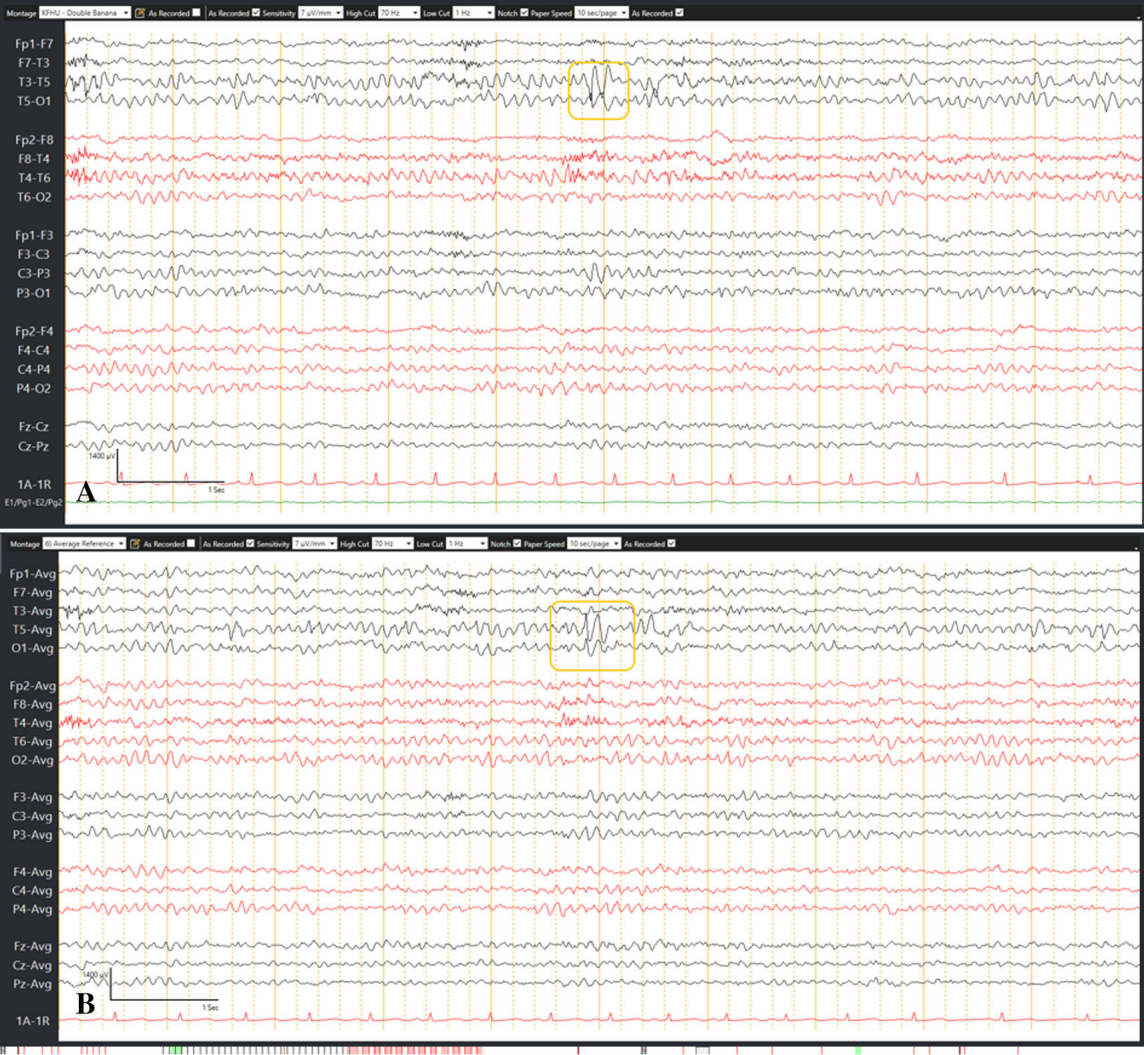
(A) Longitudinal bipolar montage, (B) Common referential montage – improvement in background rhythm and left temporal epileptic abnormality.

**Figure 3.**
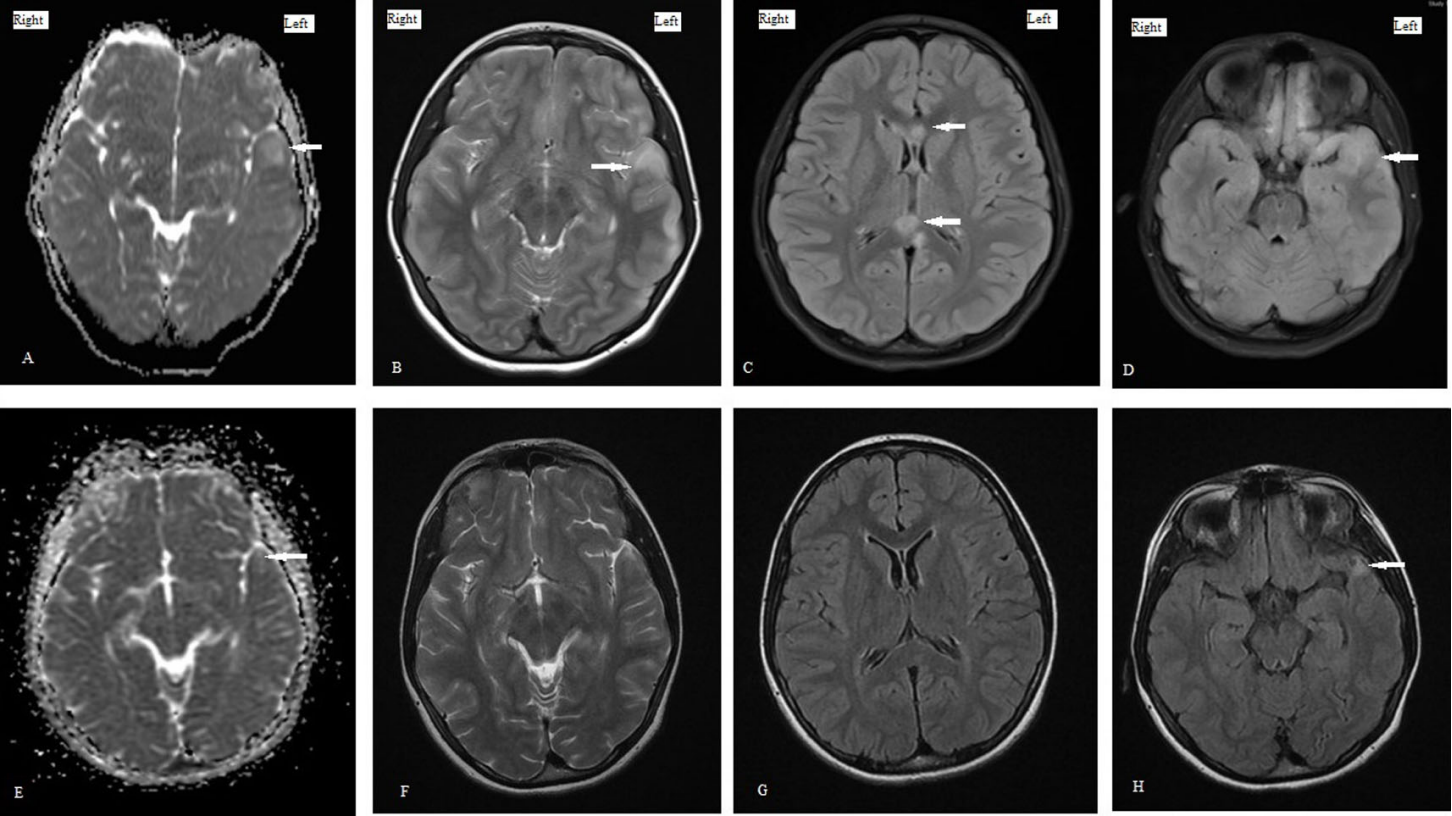
A-D: Initial MRI brain images showing changes of diffuse axonal brain injury; A - ADC sequence showing edema, B - T2 weighted images showing hyperintense left temporal lobe, C - FLAIR sequence showing hyperintense signals in corpus callosum, D - FLAIR sequence showing hyperintense left temporal lobe suggestive of edema. E-H: Follow up MRI brain images; E - ADC sequence showing residual minimal bright signal in left temporal area, F - T2 weighted sequence showing significant resolution of left temporal edema, G - FLAIR image showing resolution of changes in corpus callosum, H - FLAIR sequence showing residual hyperintense lesion in left temporal lobe.

During her follow-up visits, she was symptoms free. Brain MRI and EEG were repeated nine months after the event to determine if ASM should be discontinued. EEG detected focal epileptic discharges in the left temporal head region as shown in Figure 2, and brain MRI showed significant resolution of changes, except an abnormal focal hyperintense lesion in the left temporal area (see Figure 3).

**Figure 4.**
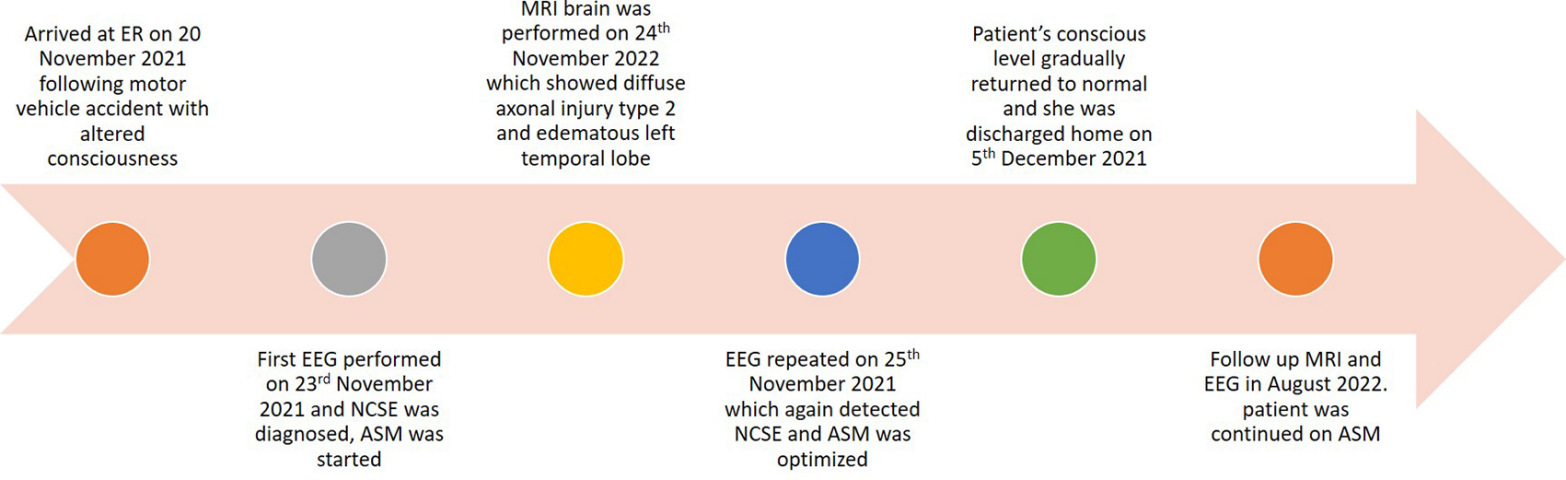
Time line of case.

Consequently, LVT was continued (the entire timeline is summarized in Figure 4).

## Outcome

One year follow-up, the patient was seizure-free with single ASM and had no symptoms suggestive of cognitive disturbance. Her neurological examination was normal and she was active in her normal routine life including academics.

## Discussion

TBI is a significant cause of preventable deaths in Saudi Arabia, and 95% are attributed to MVA. ^2^ Seizures (including NCS/NCSE) can occur in 20−30% of patients with severe TBI due to cerebral metabolic distress and hippocampal atrophy, which contribute to higher mortality rates. ^3^ However, in clinical practice, NCS following TBI may remain undiagnosed due to the lack of overt clinical manifestation and adversely affect outcomes due to the delay in treatment. ^4^ Occurrence of early NCS can have subsequent neurologic sequelae due to excitotoxic neuronal injury aggravating the injury caused by TBI. ^5^ Therefore, it is mandatory to diagnose NCSE in a timely manner to prevent significant neurological sequelae by performing continuous EEG (cEEG) monitoring in TBI victims with altered sensorium. The benefits of this protocol are supported by the findings yielded by a study involving cEEG in 16 patients with severe TBI, allowing NCS to be detected in three cases. ^6^ Similarly, seizures were detected using cEEG in 20% of examined patients, more than 50% of whom experienced NCS. ^7^

Our patient had TBI of moderate severity ^8^ and prolonged NCSE which lasted for five days. Brain MRI showed changes related to prolonged NCS, along with DAI. The follow-up EEG detected focal epileptic discharges in the left temporal region and MRI showed a small localized hyperintense lesion in the left temporal lobe which could be either a sequela of TBI or NCSE. However, the left temporal cortical edema, which could be the result of prolonged NCS, was completely resolved. The relationship between prolonged NCS and structural changes to the brain, particularly the temporal lobe, has been reported previously. ^3^^,^^9^ Vespa et al. examined cEEG findings of 140 patients with moderate to severe TBI, and detected acute post-traumatic NCS in 23% of the cohort. Moreover, their data identified NCS occurring in acute TBI to be associated with disproportionate hippocampal atrophy in long term which was greater in hippocampus ipsilateral to the Esz focus as compared to the hippocampus without seizure. ^3^ In addition, according to one case report, a patient with schizophrenia and NCSE having increased hippocampal volume in an acute setting was later found to have hippocampal atrophy. ^9^ Although NCSE is not uncommon following TBI, its association with anatomical changes leading to hippocampal atrophy in the long term is debatable, given that significant neuronal damage due to diffuse injury itself can be a cause. Thus, further research is required to better understand these phenomena. ^10^ Jorge et al., studied 37 patients with closed head injury and concluded that hippocampal volumes were significantly lower in patients with moderate to severe head injury than in patients with mild TBI. ^11^ Furthermore, an important observation is that MRI changes in patients with status epilepticus (SE) are variable and dynamic. A retrospective study investigating MRI findings in patients with SE reported that MRI changes can be diffuse or focal and can be seen in different extratemporal brain areas (brain stem, cerebellum, thalamus and basal ganglia). Importantly, the authors recommended that as these changes can evolve after one week, serial MRI should be performed to follow these neuroanatomical changes related to SE. ^12^ It is also worth noting that recent studies find MRI changes useful in diagnosing NCS, especially when EEG is inconclusive or in setups where cEEG is not available. ^13^

Patients with TBI can have various rhythmic and periodic patterns apart from diffuse slowing and Esz in their EEGs. Whether these electrographic patterns observed in patients with TBI affect functional outcomes or not is another challenging question that can be addressed by cEEG. Lee et al. studied 152 patients sustaining non-penetrating moderate to severe TBI with cEEG and observed no correlation ship between rhythmic, periodic, and ictal patterns including Esz and functional outcome at three months. However, only four patients had Esz in this study and therefore further studies including a higher number of patients with Esz are needed to evaluate its effect on outcome. Nevertheless, an independent association was established between cEEG background and functional outcome which defines the prognostic value of cEEG in patients with TBI. ^14^ In another study describing the acute physiological effect of NCS in moderate to severe TBI by cEEG and cerebral microdialysis for 7 days, ten patients with Esz were compared with ten patients without seizures. The patients with Esz as compared to non-seizure patients were found to have higher mean intracranial pressure (ICP) and a higher mean lactate/pyruvate ratio suggesting Esz to be the cause of metabolic crisis and raised ICP in addition to TBI itself. ^15^ These findings are important and emphasize the need for cEEG to detect and treat Esz timely to prevent further brain cellular injury which can be permanent. ^16^

In our patient, follow-up brain MRI showed a significant resolution of findings detected in the initial scan; however, a small abnormal hyperintense signal was persistent in the left anterior temporal region. As volumetric MRI is not available in our healthcare facility, we were unable to assess the volume loss. Still, we posit that—in addition to TBI—prolong NCSE in our patient could be a contributing factor for this finding on follow-up MRI. Although our patient did not have any clinical seizures, follow-up EEG and MRI performed nine months after the initial incident were suggestive of temporal lobe pathology with a heightened risk of temporal lobe-onset seizure. Accordingly, her ASM could not be discontinued. Routine EEG should be performed without any delay in setsup lacking the facility for cEEG. In this particular case, brain MRI was instrumental for detecting changes not only in DAI but possibly NCS as well. This case highlights our limitation of immediate EEG recording in patients with TBI upon arrival. The strength is the identification of an electrographic patterns as NCS during routine EEG recording based on Salzburg Consensus Criteria to diagnose NCSE guiding proper management. In reporting on this case, we aim to highlight the importance of cEEG monitoring in patients with TBI in whom consciousness is altered and emphasize the implementation of a consensus statement for cEEG by Herman et al. ^17^ The American Electroencephalographic Society recommends that every patient with acute brain injury having an altered level of awareness should be evaluated by cEEG to detect Esz as it can have an impact on the outcome. ^17^

## Conclusion

This case illustrates the possible association of prolonged NCSE following TBI with temporal lobe structural changes. It further emphasizes the need for immediate and cEEG monitoring in patients with TBI that present with altered sensorium. All institutes dealing with trauma cases should thus have the resources needed for emergency cEEG monitoring to avoid neurological sequelae of NCS, which may otherwise remain undiagnosed.

## Consent

We confirm that we have obtained permission from the patients’ father to use images and data included in this article.

## Data Availability

All data underlying the results are available as part of the article and no additional source data are required.
