# Supplementary material for: Case Report: Non-convulsive seizure following traumatic brain injury — a significant occurrence that needs to be considered due to potential long-term sequelae
Source: F1000Res. 2024 Jan 24;12:1155. Originally published 2023 Sep 14. [Version 2] doi: 10.12688/f1000research.135482.2 (PMC11079723; doi:10.12688/f1000research.135482.2)
Supplement: Peer review history

# Reviewer report: Reviewer response for version 2

**DOI:** 10.5256/f1000research.161327.r240429

**Published:** 2024-04-29

**Authors:** Seyed M Mirsattari

**Affiliations:** 1 Western University, London, Ontario, Canada

I approve the revisions

Are enough details provided of any physical examination and diagnostic tests, treatment given and outcomes?

Partly

Is the case presented with sufficient detail to be useful for other practitioners?

Partly

Is sufficient discussion included of the importance of the findings and their relevance to future understanding of disease processes, diagnosis or treatment?

Partly

Is the background of the case’s history and progression described in sufficient detail?

Yes

Reviewer Expertise:

Electroencephalography, neuroimaging, epilepsy, status epilepticus

I confirm that I have read this submission and believe that I have an appropriate level of expertise to confirm that it is of an acceptable scientific standard.

# Reviewer report: Reviewer response for version 2

**DOI:** 10.5256/f1000research.161327.r240428

**Published:** 2024-02-15

**Authors:** Maimoona Siddiqui

**Affiliations:** 1 Department of Neurology, Shifa International Hospital, Islamabad, Pakistan

I have reviewed the revised manuscript. All changes are incorporated.

Are enough details provided of any physical examination and diagnostic tests, treatment given and outcomes?

Partly

Is the case presented with sufficient detail to be useful for other practitioners?

Yes

Is sufficient discussion included of the importance of the findings and their relevance to future understanding of disease processes, diagnosis or treatment?

Yes

Is the background of the case’s history and progression described in sufficient detail?

Yes

Reviewer Expertise:

Epilepsy, Stroke

I confirm that I have read this submission and believe that I have an appropriate level of expertise to confirm that it is of an acceptable scientific standard.

# Reviewer report: Reviewer response for version 1

**DOI:** 10.5256/f1000research.148602.r218441

**Published:** 2023-11-30

**Authors:** Seyed M Mirsattari

**Affiliations:** 1 Western University, London, Ontario, Canada

This is an interesting case report with an important take home message about non-convulsive status epilepticus as a potential cause of immediate and delayed recovery from a traumatic brain injury. However, the EEG figure is not very convincing. Could a better figure be provided? The need for continuous EEG (cEEG) rather than serial EEGs needs to be emphasized. The authors do not explain the discrepancy between their MRI and EEG changes: EEG showed left temporal focal status epilepticus but MRI findings were bifrontal, "right" temporo-occipital and within the corpus callosum. Please clarify if you meant to say, "left temporal" lobe as your figure indicates it. You may label your MRI images with R and L to avoid confusion. Important reference: Cartagena AM, et al. Reversible and irreversible cranial MRI findings associated with status epilepticus. Epilepsy Behav 2014 Apr:33:24-30 ^1^.

Are enough details provided of any physical examination and diagnostic tests, treatment given and outcomes?

Partly

Is the case presented with sufficient detail to be useful for other practitioners?

Partly

Is sufficient discussion included of the importance of the findings and their relevance to future understanding of disease processes, diagnosis or treatment?

Partly

Is the background of the case’s history and progression described in sufficient detail?

Yes

Reviewer Expertise:

Electroencephalography, neuroimaging, epilepsy, status epilepticus

I confirm that I have read this submission and believe that I have an appropriate level of expertise to confirm that it is of an acceptable scientific standard, however I have significant reservations, as outlined above.

## References

[1] : Reversible and irreversible cranial MRI findings associated with status epilepticus. *Epilepsy Behav* .2014;33: 10.1016/j.yebeh.2014.02.003 24-30 10.1016/j.yebeh.2014.02.003 24614522

# Response

**Published:** 2023-12-05

**Authors:** Azra Zafar

**Affiliations:** Neurology, Imam Abdulrahman Bin Faisal University, Dammam, Eastern Province, Saudi Arabia

We are grateful to the reviewer for reviewing our paper and giving valuable suggestions. We have responded to each comment accordingly and hope it will be satisfying.

 This is an interesting case report with an important take home message about non-convulsive status epilepticus as a potential cause of immediate and delayed recovery from a traumatic brain injury. However, the EEG figure is not very convincing. Could a better figure be provided?

 Response; Thanks for your comment, I have added another figure here with more explanation.

 Description will be added in the manuscript.

 Background activity consisted mainly of diffuse theta activity. Frequent 2-3 Hz left hemispheric ictal delta activity with spatio-temporal evolution was recorded. The activity starting from the left hemisphere propagated after a few seconds to involve the right hemisphere. In, a 30-minute EEG recording, 7 events lasting for 7-20 seconds were recorded .sharp wave activity was also recorded over the left fronto-central and temporal head region. Some spells of these epileptiform patterns were associated with subtle clinical signs which were left-hand automatism and screaming indicating electro-clinical seizures.

 Response; Kindly see the epochs here.

 1a- Beginning of electrographic seizure; rhythmic slow waves showing propagation to right cerebral hemisphere.

 1b- Continuation and termination of electrographic seizure.

 We can further add follow up EEG shown below as Figure 3 to document improvement of background rhythm and left temporal epileptic abnormality.

 The need for continuous EEG (cEEG) rather than serial EEGs needs to be emphasized.

 Response; done as suggested and more references have been added to emphasize the need of cEEG.

 Whether the electrographic patterns observed in patients with TBI affect functional outcome or not is another challenging question that can be addressed by cEEG. Lee et al. studied 152 patients sustaining non-penetrating moderate to severe TBI with cEEG and observed no correlation ship between rhythmic, periodic, and ictal patterns including Esz and functional outcome at three months. However, only four patients had Esz in this study and therefore further studies including a higher number of patients with Esz are needed to evaluate its effect on outcome. Nevertheless, an independent association was established between cEEG background and functional outcome which defines the prognostic value of cEEG in patients with TBI [Lee H 2019].

 Lee H, Mizrahi MA, Hartings JA, Sharma S, Pahren L, Ngwenya LB, Moseley BD, Privitera M, Tortella FC, Foreman B. Continuous Electroencephalography After Moderate to Severe Traumatic Brain Injury. Crit Care Med. 2019 Apr;47(4):574-582. doi: 10.1097/CCM.0000000000003639. PMID: 30624278; PMCID: PMC6639805.

 In another study describing the acute physiological effect of NCS in moderate to severe TBI by cEEG and cerebral microdialysis for 7 days, ten patients with Esz were compared with ten patients without seizures. The patients with Esz as compared to non-seizure patients were found to have higher mean intracranial pressure (ICP) and a higher mean lactate/ pyruvate ratio suggesting Esz to be the cause of metabolic crisis and raised ICP in addition to TBI itself [Vespa PM 2007]. These findings are important and emphasize the need for cEEG to detect and treat Esz timely to prevent further brain cellular injury which can be permanent [Hirsch LJ 2008].

 Vespa PM, Miller C, McArthur D, Eliseo M, Etchepare M, Hirt D, Glenn TC, Martin N, Hovda D. Nonconvulsive electrographic seizures after traumatic brain injury result in a delayed, prolonged increase in intracranial pressure and metabolic crisis. Crit Care Med. 2007 Dec;35(12):2830-6. PMID: 18074483; PMCID: PMC4347945.

 Hirsch LJ. Nonconvulsive Seizures in Traumatic Brain Injury: What you Don’t See Can Hurt You. *Epilepsy Currents*. 2008;8(4):97-99. doi: 10.1111/j.1535-7511.2008.00254.x

 In reporting on this case, we aim is to highlight the importance of cEEG monitoring in patients with TBI in whom consciousness is altered and emphasize the implementation of a consensus statement for cEEG by Herman et al. The American Electroencephalographic Society recommends that every patient with acute brain injury having an altered level of awareness should be evaluated by cEEG to detect ESz as it can have an impact on the outcome. [Herman ST 2015]. 

 Herman ST, Abend NS, Bleck TP, et al. Consensus statement on continuous EEG in critically ill adults and children, part I: indications. Journal of clinical neurophysiology : official publication of the American Electroencephalographic Society. 2015. April;32(2):87–95.

 The authors do not explain the discrepancy between their MRI and EEG changes: EEG showed left temporal focal status epilepticus but MRI findings were bifrontal, "right" temporo-occipital and within the corpus callosum. Please clarify if you meant to say, "left temporal" lobe as your figure indicates it. You may label your MRI images with R and L to avoid confusion.

 Response; thanks for pointing out this mistake, it was left temporal lobe as mentioned in the figure. We will correct it in revised manuscript.

 We have corrected the EEG findings in the text as well. Kindly see the above description for EEG. It was left hemispheric delta activity propagating to the right.

 We have added R and L in MRI images.

 Important reference: Cartagena AM, et al. Reversible and irreversible cranial MRI findings associated with status epilepticus. Epilepsy Behav 2014 Apr:33:24-30 ^1^.

 Response; thank you for mentioning. This will be added in the discussion in the revised manuscript.

 Previous studies have reported various MRI changes in SE. A retrospective study investigating MRI findings in patients with SE reported that MRI changes can be either diffuse or focal and can be seen in different extratemporal brain areas including the brain stem, cerebellum, thalamus and basal ganglia. Importantly, the authors recommended that as these changes can evolve after one week, serial MRI should be performed to follow these neuroanatomical changes related to SE and reversibility. [Cartagena AM].

 Cartagena AM, Young GB, Lee DH, Mirsattari SM: Reversible and irreversible cranial MRI findings associated with status epilepticus.Epilepsy Behav. 2014; **33**: 24-30 PubMed Abstract | Publisher Full Text

 It is also worth noting that recent studies find MRI changes useful in diagnosing NCS, especially when EEG is inconclusive or in setups where cEEG is not available [Gelisse P].

 Gelisse P, Genton P, Crespel A, Lefevre PH. Will MRI replace the EEG for the diagnosis of nonconvulsive status epilepticus, especially focal? Rev Neurol. 2021; 177:359-69. doi: 10.1016/j.neurol.2020.09.005. Epub 2021 Jan 22. PMID: 33487411.

  

- **Is the background of the case’s history and progression described in sufficient detail?**

Yes

 Thanks for the response

- **Are enough details provided of any physical examination and diagnostic tests, treatment given and outcomes?**

Partly

 Response; further details related to examination, outcome, and follow up EEG will be included in the revised manuscript

 The patient was agitated with a fluctuating level of consciousness at the time of presentation and did not follow commands. Cranial nerves were intact. Tone, power and deep tendon reflexes were normal. There were no signs of meningism.

 After starting the second ASM, the patient’s consciousness improved and returned to baseline. Patient became oriented to time, place, and person and had intact language functions. Patient was able to walk independently and had normal motor, cerebellar, and sensory system examination before discharge.

 At one year follow-up, the patient was perfectly normal and had no clinical seizures or cognitive disturbances. The follow-up EEG detected left temporal epileptic abnormality. MRI images included.

  

- **Is sufficient discussion included of the importance of the findings and their relevance to future understanding of disease processes, diagnosis or treatment?**

Partly

 Response; We have further elaborated the discussion, hope that you will find it satisfactory now.

- **Is the case presented with sufficient detail to be useful for other practitioners?**

Partly

 Response; we have added more details.

**References**

 1. Cartagena AM, Young GB, Lee DH, Mirsattari SM: Reversible and irreversible cranial MRI findings associated with status epilepticus.Epilepsy Behav. 2014; **33**: 24-30 PubMed Abstract | Publisher Full Text

 Response; added

 Moreover, a retrospective study investigating MRI findings in patients with status epilepticus reported that MRI changes can be diffuse or focal and can be seen in different extratemporal brain areas (brain stem, cerebellum, thalamus and basal ganglia). Importantly, the authors recommended that as these changes can evolve after one week, serial MRI should be performed to follow these neuroanatomical changes related to SE. [Cartagena AM].

 Data have identified NCS occurring in acute TBI to be associated with disproportionate hippocampal atrophy in long term which is greater in Hippocampus ipsilateral to the Esz focus as compared to the hippocampus without seizure. [Vespa PM 2010]

 Vespa PM, McArthur DL, Xu Y, et al.: Nonconvulsive seizures after traumatic brain injury are associated with hippocampal atrophy. Neurology. 2010; **75**: 792–798. PubMed Abstract | Publisher Full Text | Free Full Text

# Response

**Published:** 2023-12-10

**Authors:** Azra Zafar

**Affiliations:** Neurology, Imam Abdulrahman Bin Faisal University, Dammam, Eastern Province, Saudi Arabia

We are grateful to the reviewer for reviewing our paper and giving valuable suggestions. We have responded to each comment accordingly and hope it will be satisfying.

 This is an interesting case report with an important take home message about non-convulsive status epilepticus as a potential cause of immediate and delayed recovery from a traumatic brain injury. However, the EEG figure is not very convincing. Could a better figure be provided?

**Response**; Thanks for your comment, I have added another figure here with more explanation.

 Description will be added in the manuscript.

 Background activity consisted mainly of diffuse theta activity. Frequent 2-3 Hz left hemispheric ictal delta activity with spatio-temporal evolution was recorded. The activity starting from the left hemisphere propagated after a few seconds to involve the right hemisphere. In, a 30-minute EEG recording, 7 events lasting for 7-20 seconds were recorded .sharp wave activity was also recorded over the left fronto-central and temporal head region. Some spells of these epileptiform patterns were associated with subtle clinical signs which were left-hand automatism and screaming indicating electro-clinical seizures.

**Response**; Kindly see the epochs here.

 1a- Beginning of electrographic seizure; rhythmic slow waves showing propagation to right cerebral hemisphere.

 1b- Continuation and termination of electrographic seizure.

 We can further add follow up EEG shown below as Figure 3 to document improvement of background rhythm and left temporal epileptic abnormality.

 The need for continuous EEG (cEEG) rather than serial EEGs needs to be emphasized.

**Response**; done as suggested and more references have been added to emphasize the need of cEEG.

 Whether the electrographic patterns observed in patients with TBI affect functional outcome or not is another challenging question that can be addressed by cEEG. Lee et al. studied 152 patients sustaining non-penetrating moderate to severe TBI with cEEG and observed no correlation ship between rhythmic, periodic, and ictal patterns including Esz and functional outcome at three months. However, only four patients had Esz in this study and therefore further studies including a higher number of patients with Esz are needed to evaluate its effect on outcome. Nevertheless, an independent association was established between cEEG background and functional outcome which defines the prognostic value of cEEG in patients with TBI [Lee H 2019].

 Lee H, Mizrahi MA, Hartings JA, Sharma S, Pahren L, Ngwenya LB, Moseley BD, Privitera M, Tortella FC, Foreman B. Continuous Electroencephalography After Moderate to Severe Traumatic Brain Injury. Crit Care Med. 2019 Apr;47(4):574-582. doi: 10.1097/CCM.0000000000003639. PMID: 30624278; PMCID: PMC6639805.

 In another study describing the acute physiological effect of NCS in moderate to severe TBI by cEEG and cerebral microdialysis for 7 days, ten patients with Esz were compared with ten patients without seizures. The patients with Esz as compared to non-seizure patients were found to have higher mean intracranial pressure (ICP) and a higher mean lactate/ pyruvate ratio suggesting Esz to be the cause of metabolic crisis and raised ICP in addition to TBI itself [Vespa PM 2007]. These findings are important and emphasize the need for cEEG to detect and treat Esz timely to prevent further brain cellular injury which can be permanent [Hirsch LJ 2008].

 Vespa PM, Miller C, McArthur D, Eliseo M, Etchepare M, Hirt D, Glenn TC, Martin N, Hovda D. Nonconvulsive electrographic seizures after traumatic brain injury result in a delayed, prolonged increase in intracranial pressure and metabolic crisis. Crit Care Med. 2007 Dec;35(12):2830-6. PMID: 18074483; PMCID: PMC4347945.

 Hirsch LJ. Nonconvulsive Seizures in Traumatic Brain Injury: What you Don’t See Can Hurt You. *Epilepsy Currents*. 2008;8(4):97-99. doi: 10.1111/j.1535-7511.2008.00254.x

 In reporting on this case, we aim is to highlight the importance of cEEG monitoring in patients with TBI in whom consciousness is altered and emphasize the implementation of a consensus statement for cEEG by Herman et al. The American Electroencephalographic Society recommends that every patient with acute brain injury having an altered level of awareness should be evaluated by cEEG to detect ESz as it can have an impact on the outcome. [Herman ST 2015]. 

 Herman ST, Abend NS, Bleck TP, et al. Consensus statement on continuous EEG in critically ill adults and children, part I: indications. Journal of clinical neurophysiology : official publication of the American Electroencephalographic Society. 2015. April;32(2):87–95.

 The authors do not explain the discrepancy between their MRI and EEG changes: EEG showed left temporal focal status epilepticus but MRI findings were bifrontal, "right" temporo-occipital and within the corpus callosum. Please clarify if you meant to say, "left temporal" lobe as your figure indicates it. You may label your MRI images with R and L to avoid confusion.

**Response**; thanks for pointing out this mistake, it was left temporal lobe as mentioned in the figure. We will correct it in revised manuscript.

 We have corrected the EEG findings in the text as well. Kindly see the above description for EEG. It was left hemispheric delta activity propagating to the right.

 We have added R and L in MRI images.

 Important reference: Cartagena AM, et al. Reversible and irreversible cranial MRI findings associated with status epilepticus. Epilepsy Behav 2014 Apr:33:24-30 ^1^.

**Response**; thank you for mentioning. This will be added in the discussion in the revised manuscript.

 Previous studies have reported various MRI changes in SE. A retrospective study investigating MRI findings in patients with SE reported that MRI changes can be either diffuse or focal and can be seen in different extratemporal brain areas including the brain stem, cerebellum, thalamus and basal ganglia. Importantly, the authors recommended that as these changes can evolve after one week, serial MRI should be performed to follow these neuroanatomical changes related to SE and reversibility. [Cartagena AM].

 Cartagena AM, Young GB, Lee DH, Mirsattari SM: Reversible and irreversible cranial MRI findings associated with status epilepticus.Epilepsy Behav. 2014; **33**: 24-30 PubMed Abstract | Publisher Full Text

 It is also worth noting that recent studies find MRI changes useful in diagnosing NCS, especially when EEG is inconclusive or in setups where cEEG is not available [Gelisse P].

 Gelisse P, Genton P, Crespel A, Lefevre PH. Will MRI replace the EEG for the diagnosis of nonconvulsive status epilepticus, especially focal? Rev Neurol. 2021; 177:359-69. doi: 10.1016/j.neurol.2020.09.005. Epub 2021 Jan 22. PMID: 33487411.

  

- **Is the background of the case’s history and progression described in sufficient detail?**

Yes

 Thanks for the response

- **Are enough details provided of any physical examination and diagnostic tests, treatment given and outcomes?**

Partly

**Response**; further details related to examination, outcome, and follow up EEG will be included in the revised manuscript

 The patient was agitated with a fluctuating level of consciousness at the time of presentation and did not follow commands. Cranial nerves were intact. Tone, power and deep tendon reflexes were normal. There were no signs of meningism.

 After starting the second ASM, the patient’s consciousness improved and returned to baseline. Patient became oriented to time, place, and person and had intact language functions. Patient was able to walk independently and had normal motor, cerebellar, and sensory system examination before discharge.

 At one year follow-up, the patient was perfectly normal and had no clinical seizures or cognitive disturbances. The follow-up EEG detected left temporal epileptic abnormality. MRI images included.

  

- **Is sufficient discussion included of the importance of the findings and their relevance to future understanding of disease processes, diagnosis or treatment?**

Partly

**Response**; We have further elaborated the discussion, hope that you will find it satisfactory now.

- **Is the case presented with sufficient detail to be useful for other practitioners?**

Partly

**Response**; we have added more details.

**References**

 1. Cartagena AM, Young GB, Lee DH, Mirsattari SM: Reversible and irreversible cranial MRI findings associated with status epilepticus.Epilepsy Behav. 2014; **33**: 24-30 PubMed Abstract | Publisher Full Text

 Response; added

 Moreover, a retrospective study investigating MRI findings in patients with status epilepticus reported that MRI changes can be diffuse or focal and can be seen in different extratemporal brain areas (brain stem, cerebellum, thalamus and basal ganglia). Importantly, the authors recommended that as these changes can evolve after one week, serial MRI should be performed to follow these neuroanatomical changes related to SE. [Cartagena AM].

 Data have identified NCS occurring in acute TBI to be associated with disproportionate hippocampal atrophy in long term which is greater in Hippocampus ipsilateral to the Esz focus as compared to the hippocampus without seizure. [Vespa PM 2010]

 Vespa PM, McArthur DL, Xu Y, et al.: Nonconvulsive seizures after traumatic brain injury are associated with hippocampal atrophy. Neurology. 2010; **75**: 792–798. PubMed Abstract | Publisher Full Text | Free Full Text

 Figures , 1a,1b electrographic seizure and figure 3; follow up EEG (please see the figures linked below):

Figure 1a

Figure 1b

Figure 3

# Reviewer report: Reviewer response for version 1

**DOI:** 10.5256/f1000research.148602.r206929

**Published:** 2023-10-16

**Authors:** Maimoona Siddiqui

**Affiliations:** 1 Department of Neurology, Shifa International Hospital, Islamabad, Pakistan

Interesting case, agreed that NCSE is an under recognized entity.

 Can the author explain the meaning of "medically free" mentioned in first line? I think its need rephrasing.

 It would be better if author elaborate neurological findings like fundus examination, signs of meningism at admission and during the course of illness.

 What criteria of NCSE was followed in this case? As EEG showed 8 seconds of focal epileptiform discharges. 

 It would be better to upload a more detail EEG epoch as it is the most relevant investigation.

Are enough details provided of any physical examination and diagnostic tests, treatment given and outcomes?

Partly

Is the case presented with sufficient detail to be useful for other practitioners?

Yes

Is sufficient discussion included of the importance of the findings and their relevance to future understanding of disease processes, diagnosis or treatment?

Yes

Is the background of the case’s history and progression described in sufficient detail?

Yes

Reviewer Expertise:

Epilepsy, Stroke

I confirm that I have read this submission and believe that I have an appropriate level of expertise to confirm that it is of an acceptable scientific standard, however I have significant reservations, as outlined above.

# Response

**Published:** 2023-10-17

**Authors:** Azra Zafar

**Affiliations:** Neurology, Imam Abdulrahman Bin Faisal University, Dammam, Eastern Province, Saudi Arabia

We thank the reviewer for reviewing our paper and giving useful suggestions. We have responded to each comment accordingly and hope it will be satisfying.

 1-Can the author explain the meaning of "medically free" mentioned in first line? I think its need rephrasing.

 Response; It will be rephrased as suggested in revised manuscript;

 A 13-years old student without any prior medical illnesses was brought to the King Fahd Hospital of the University (KFHU) after sustaining a motor vehicle accident (MVA) as a car passenger.

 2-It would be better if author elaborate neurological findings like fundus examination, signs of meningism at admission and during the course of illness.

 Response; will be added; Patient was agitated with fluctuating level of consciousness at time of presentation and did not follow commands.   Cranial nerves were intact, fundus examination was attempted but failed as patient could not cooperate due to her clinical state. Tone, power and deep tendon reflexes were normal. There were no signs of meningism.

 After starting second ASM, patient’s consciousness improved and returned to baseline. Patient became oriented to time, place, and person and had intact language functions. Patient was able to walk independently and had normal motor, cerebellar, and sensory system examination before discharge.  

 What criteria of NCSE was followed in this case? As EEG showed 8 seconds of focal epileptiform discharges. 

 Response; Salzburg Consensus Criteria were applied to diagnose NCSE.

 Reference will be added.

1. Beniczky S, Hirsch LJ, Kaplan PW, Pressler R, Bauer G, Aurlien H, et al. Unified EEG terminology and criteria for nonconvulsive status epilepticus. Epilepsia. 2013; 54 (Suppl. 6):28–9.

These details will be mentioned in revised manuscript; EEG fulfilled the Salzburg Consensus Criteria for NCSE; RTDA (rhythmic theta or delta activity) with spatiotemporal evolution/ and subtle clinical signs.

 Detailed EEG report has been added.

 Background activity consisted mainly of diffuse theta activity. Frequent 2-3 Hz left hemispheric ictal delta activity with spatio-temporal evolution was recorded. The activity starting from left hemisphere and propagated after few seconds to involve the right hemisphere. In, a 30 minutes EEG recording, 7 events lasting for 7-20 seconds were recorded .Occasional sharp wave activity was also recorded over the left fronto-central head region with maximal negativity at C3. Some spells of these epileptiform pattern were associated with subtle clinical signs which were left hand automatism and screaming indicating electro-clinical seizures.

 It would be better to upload a more detail EEG epoch as it is the most relevant investigation.

 Response; Kindly see the epochs here.



 Figure 1a



 Figure 1b

 1a- Beginning of electrographic seizure; rhythmic slow waves showing propagation to right cerebral hemisphere.

 1b- Continuation and termination of electrographic seizure.

 We can further add follow up EEG shown below as Figure 3 to document improvement of background rhythm and left temporal epileptic abnormality.
